# The Complete Mitogenome of *Alternaria brassicae* Causing Black Spot Disease on Rapeseed-Mustard

**DOI:** 10.3390/jof12090648

**Published:** 2026-09-01

**Authors:** Zhiyuan Mao, Guangyuan Bai, Ke Wang, Yang Yang

**Affiliations:** 1 Beijing National Laboratory for Condensed Matter Physics, Institute of Physics, Chinese Academy of Sciences, Beijing 100190, China; 2 College of Horticulture and Landscape Architecture, Northeast Agricultural University, Harbin 150030, China; 3 Key Laboratory of Biology and Genetic Improvement of Horticulture Crops (Northeast Region), Ministry of Agriculture and Rural Affairs, Northeast Agricultural University, Harbin 150030, China

**Keywords:** *Alternaria brassicae*, mitochondrial genome, phylogenetic analysis, black spot

## Abstract

*Alternaria brassicae* is a destructive fungal pathogen that causes black spot disease on rapeseed-mustard and other cruciferous crops, leading to significant yield losses worldwide. Despite its economic importance, the mitochondrial genome of *A. brassicae* has remained unreported. De novo assembly of publicly available PacBio HiFi reads using the HiMT toolkit generated a circular mitochondrial DNA molecule of 50,713 bp with an average sequencing coverage of 309.0×. The genome encodes 19 protein-coding genes, comprising 12 named mitochondrial genes (*atp6*, *cox1*, *cox3*, *cob*, *nad1*, *nad2*, *nad3*, *nad4*, *nad4L*, *nad5*, *nad6*, and *rps3*) and seven intronic open reading frames (*lagli_0–lagli_5* and *giy*), two rRNA genes (*rrnL*, *rrnS*), and 37 tRNA genes, with an overall GC content of 29.3%. Intron-containing structures were predicted in the *nad4*, *nad4L*, *nad5*, *cox3*, *cob*, and *cox1* genes; these predictions were not validated with transcript data. A total of 33 simple sequence repeat (SSR) loci were detected, predominantly A/T mononucleotide repeats, with a density of one SSR per 1.52 kb. Tandem repeats clustered mainly in two regions (33–40 kb and 45–50 kb), and interspersed repeat analysis revealed 45.83% inverted, 30.28% complementary, 12.22% direct, and 11.67% palindromic repeats. Maximum-likelihood phylogenetic analysis of 22 mitogenomes placed *A. brassicae* (CNSA: CNP0008733) as sister to *A. burnsii* with 65% bootstrap support; the deeper grouping with *A. gossypina* had 25% support. Compared with the published *A. tenuissima* mitogenome (57,475 bp, 34 tRNAs, and two rRNAs), the present assembly is 6762 bp shorter and contains three additional tRNAs. This first complete mitogenomic resource for *A. brassicae* provides a valuable foundation for future studies on population genetics, molecular diagnostics, and evolutionary dynamics, aiding the development of improved management strategies for black spot diseases in cruciferous crops.

## 1. Introduction

Cruciferous crops, encompassing oilseed rape (*Brassica napus* L.), Chinese cabbage (*Brassica rapa* ssp. *pekinensis*), cabbage (*Brassica oleracea* var. *capitata*), and radish (*Raphanus sativus* L.), represent vital sources of edible oil, dietary fiber, and essential nutrients globally [1]. Among the diverse phytopathogenic threats confronting Brassicaceae agriculture, *Alternaria brassicae* (Berk.) Sacc. stands out as a destructive fungal pathogen belonging to the class Dothideomycetes, order Pleosporales, and family Pleosporaceae [2]. This necrotrophic fungus is the primary causal agent of black spot disease (also termed Alternaria blight or leaf spot), a devastating foliar and reproductive disease that severely impairs crop health, yield stability, and commodity quality worldwide [3].

The infection cycle of *A. brassicae* initiates with the germination of airborne or seed-borne conidia on susceptible host tissues, followed by appressorium formation and direct penetration through stomata or mechanical cuticular micro-wounds [4]. Symptoms typically manifest as dark brown to black necrotic lesions encircled by prominent chlorotic halos on leaves, stems, and seed pods (siliques). As lesions expand and coalesce, infected plants suffer severe physiological impairments, including premature leaf senescence, accelerated defoliation, restricted photosynthetic efficiency, and silique shattering prior to harvest [5]. Seed pod infections are particularly detrimental to oilseed rape production, leading to premature pod dehiscence, seed shriveling, reduced seed weight, and significant deterioration in seed oil composition, characterized by elevated free fatty acid levels and diminished triacylglycerol yields [6]. In severe disease epidemics, yield losses in oilseed rape and leafy crucifers range from 15% to over 50%, inflicting substantial economic damages on global agricultural supply chains [7].

In recent years, the epidemiological landscape of *A. brassicae* has undergone noticeable shifts. Temperature and relative humidity strongly influence sporulation of *A. brassicae*; sporulation has been reported at 18–24 °C under relative humidity above 91.5% [8]. Infected crop debris can retain viable *A. brassicae* inoculum after harvest and can therefore serve as a source of infection within and between crops [9]. Alternaria blight remains an important and widely distributed disease of rapeseed-mustard and other crucifers [10]. Despite integrated disease management efforts relying on synthetic chemical fungicides (e.g., quinone outside inhibitors [QoIs] and demethylation inhibitors [DMIs]) and cultural practices, effective disease control remains challenging [11]. The limited availability of high-level genetic resistance cultivars within commercial germplasm pools, combined with the evolutionary risk of fungicide resistance development under persistent chemical selection pressure, underscores the urgency of elucidating the molecular genetics, evolutionary adaptations, and pathogenic mechanisms of *A. brassicae* [12].

Mitochondria are indispensable eukaryotic organelles responsible for cellular respiration, adenosine triphosphate (ATP) generation via oxidative phosphorylation (OXPHOS), reactive oxygen species (ROS) homeostasis, lipid biosynthesis, and programmed cell death [13]. In phytopathogenic fungi, functional mitochondria are tightly linked to metabolic flexibility, vegetative growth, stress tolerance, conidiation, host infection, and virulence determination [14]. The fungal mitochondrial genome (mitogenome) typically exists as a circular or linear double-stranded DNA molecule that encodes core proteins involved in the electron transport chain (e.g., subunits of apocytochrome b, cytochrome c oxidase, and NADH dehydrogenase), ATP synthase complexes, as well as essential ribosomal RNAs (rRNAs) and transfer RNAs (tRNAs) [15].

Despite sharing a conserved set of core metabolic genes, fungal mitogenomes exhibit remarkable architectural plasticity, displaying extensive variation in genome size, gene order (synteny), intron composition, intergenic spacer length, and repeat dynamics [16]. Variations in mitogenome size are largely driven by the expansion of non-coding intergenic regions and the proliferation of mobile genetic elements, particularly Group I and Group II introns, as well as intronic open reading frames (ORFs) encoding homing endonucleases (such as LAGLIDADG and GIY-YIG motifs) [17]. Owing to their maternal or uniparental inheritance, high copy number per cell, relatively low rate of intermolecular recombination compared to nuclear DNA, and accelerated nucleotide substitution rates, mitogenomes serve as ideal molecular markers for evolutionary biology, comparative genomics, population genetics, and species identification in fungal phytopathology [18]. Furthermore, because mitochondrial electron transport chain complexes (e.g., Complex III) represent primary target sites for major agricultural fungicides like QoIs, characterizing mitochondrial gene structures and variation is critical for monitoring drug target mutations and understanding fungicide resistance evolution [19].

Within the genus *Alternaria*, nuclear genome sequencing projects have advanced our understanding of taxonomy, host-selective toxin (HST) biosynthetic gene clusters, and comparative pathogenicity [20]. However, mitochondrial genomic resources within this large genus remain unequally distributed. While partial nuclear ribosomal gene regions (e.g., *ITS*, *RPB2*, *ALT1*, *GAPDH*) have been widely used for species identification, the complete organellar architecture and evolutionary trajectory of *A. brassicae* remain uncharacterized. The absence of a complete, high-resolution mitochondrial genome assembly for *A. brassicae* represents a major gap in understanding organelle evolution, respiratory chain composition, and mitogenomic synteny across Dothideomycete plant pathogens.

To address this knowledge gap, the present study utilizes third-generation PacBio High-Fidelity (HiFi) long-read sequencing technology to perform the first de novo assembly, comprehensive annotation, and structural characterization of the complete mitochondrial genome of *A. brassicae* isolated from oilseed rape black spot lesions. We conduct detailed analyses of its gene content, intron insertion sites, repeat element composition. Furthermore, through phylogenomic reconstruction based on concatenated mitochondrial core protein-coding genes across 22 representative fungal species, we clarify the precise evolutionary position of *A. brassicae* within the genus *Alternaria* and the order Pleosporales. The complete mitogenome sequence presented here provides a foundational genomic resource for future studies on population genetics, molecular diagnostics, mitochondrial inheritance, and fungicide resistance management in cruciferous crop protection.

## 2. Materials and Methods

### 2.1. Raw Sequencing Data Acquisition and Quality Assessment

In this study, we retrieved publicly available third-generation PacBio HiFi sequencing data from NCBI SRA (SRR32051149); the authors did not generate the sequencing data to perform the first de novo assembly, comprehensive annotation, and phylogenomic analysis of the complete mitochondrial genome of *A. brassicae*. Quality inspection of the downloaded raw dataset was performed using FastQC (v0.11.9; Babraham Institute, Cambridge, UK). Read filtering was conducted using Filtlong (v0.2.1) to retain high-accuracy HiFi reads with a minimum quality score of Q ≥ 20 and a minimum read length of 1000 bp. The filtered dataset featured an average read length ranging from 10 kb to 15 kb, which was subsequently utilized for organelle genome extraction and assembly.

### 2.2. De Novo Assembly and Circularization of the Mitogenome

To isolate and assemble the mitochondrial genome from the whole-genome long-read dataset, the specialized organelle assembly toolkit HiMT (v1.0;) [21] was employed. The HiMT pipeline utilizes an optimized overlap layout consensus (OLC) algorithm tailored specifically for PacBio HiFi reads, effectively segregating mitochondrial reads from nuclear genomic background based on k-mer depth variations and sequence similarity against organellar reference databases. The primary assembly graph was generated using dynamic overlap thresholds. Graph topology and contig ends were inspected to confirm circularization by detecting terminal overlapping redundancy, and one copy of the overlap was trimmed before annotation.

### 2.3. Genome Annotation and Gene Curation

Preliminary automated annotation of the circular *A. brassicae* mitogenome was performed using the MITOS2 web server (https://mitos2.bioinf.uni-leipzig.de; accessed on 10 July 2026) [22] and MFannot pipeline, applying the fungal mitochondrial genetic code. Protein-coding genes (PCGs) were manually validated and curated by comparing predicted open reading frames (ORFs) against non-redundant protein databases (NCBI nr) using BLASTp v2.12.0+ (NCBI, Bethesda, MD, USA) searches. Canonical start codons (AUG) and alternative initiation codons (e.g., UUG, GUG), as well as termination codons (UAA, UAG), were confirmed manually via alignment with homologous mitochondrial proteins from closely related *Alternaria* species (*Alternaria alternata*, *Alternaria solani*, and *Alternaria destruens*).

Transfer RNA (tRNA) genes were identified using tRNAscan-SE (v2.0.11; Lowe Lab, University of California, Santa Cruz, CA, USA) [23] in organellar search mode with default cutoff parameters. Ribosomal RNA (rRNA) genes—specifically the small subunit ribosomal RNA (rns) and large subunit ribosomal RNA (rnl)—were delineated by combining RNAmmer (v1.2; Technical University of Denmark, Lyngby, Denmark) [24] predictions with local BLASTn v2.12.0+ alignments against reference Dothideomycete mitochondrial rRNA sequences.

Intron–exon boundaries within split genes were determined by aligning intron-containing sequences with intronless homologous protein sequences from related species. Intron types (Group I and Group II) were classified according to secondary structure characteristics and sequence motifs. Intronic open reading frames encoding LAGLIDADG or GIY-YIG homing endonucleases were annotated through NCBI Conserved Domain Database (CDD) searches [25]. These intron–exon assignments were predicted in silico and were not validated by RNA-seq or RT-PCR.

The complete circular genome map of *A. brassicae* was rendered using the Proksee online platform (https://proksee.ca; accessed on 10 July 2026; University of Alberta, Edmonton, AB, Canada) utilizing the CGView rendering engine. The visual map displays major genomic features, including forward- and reverse-strand PCGs, tRNAs, rRNAs, and predicted introns [26].

### 2.4. Repeat Structure Analysis

Simple Sequence Repeats (SSRs), or microsatellites, were identified across the entire mitogenome using the MISA (Leibniz Institute of Plant Genetics and Crop Plant Research, Gatersleben, Germany) [27]. Parameters for minimum motif repetitions were set as follows: 10 for mono-nucleotide repeats, 5 for di-nucleotide repeats, 4 for tri-nucleotide repeats, and 3 each for tetra-, penta-, and hexa-nucleotide repeats.

Tandem repeat structures were detected using Tandem Repeats Finder (TRF v4.09; Boston University, Boston, MA, USA) [28] with default alignment parameters (match score = 2, mismatch penalty = 7, indel penalty = 7, matching probability = 80, minscore = 50, maximum period size = 500). Dispersed repeat elements—including forward (direct), reverse, complement, and palindromic repeats—were evaluated via self-comparison BLASTn v2.12.0+ searches and the BIBIServ2 Repeat Finder platform (https://bibiserv.cebitec.uni-bielefeld.de/repeatfinder; accessed on 10 July 2026) [29].

### 2.5. Phylogenomic Reconstruction and Comparative Mitogenomics

To resolve the evolutionary lineage and taxonomic position of *A. brassicae*, comparative mitogenomic analyses were conducted using the new assembly and 21 published fungal mitochondrial genomes (22 taxa total) downloaded from NCBI GenBank (https://www.ncbi.nlm.nih.gov/genbank/; accessed on 10 July 2026). The sampling comprised seven *Alternaria* taxa (including *A. brassicae*), 13 other Pleosporales taxa, and two external *Ceratocystiopsis* taxa from Ophiostomatales. *Acrocalymma* sp. (PP151286) was used to root the displayed tree. Maximum-likelihood inference was performed in MEGA 11 (Pennsylvania State University, University Park, PA, USA) [30], and the sampling included published *Exserohilum* mitochondrial sequences [31].

The 12 mitochondrial PCGs shared across the sampled taxa (*atp6*, *cob*, *cox1*, *cox3*, *nad1*, *nad2*, *nad3*, *nad4*, *nad4L*, *nad5*, *nad6*, and *rps3*) were individually aligned at the amino acid level using MAFFT (v7.490) [32] under the L-INS-i algorithm, trimmed using TrimAl (v1.4.1; Spanish National Cancer Research Centre, Madrid, Spain), and subsequently back-translated to their corresponding nucleotide coding sequences (CDS) to preserve codon alignment [33]. The concatenated nucleotide matrix was subjected to model testing in ModelTest-NG, identifying GTR + G + I as the optimal nucleotide substitution model for Maximum Likelihood (ML) tree construction in MEGA 11.

Trimmed single-gene alignments were concatenated into a master multi-gene alignment matrix using PhyloSuite (v1.2.3; Institute of Zoology, Chinese Academy of Sciences, Beijing, China) [34]. Model selection was conducted using ModelTest-NG [35] based on the Akaike Information Criterion (AIC). Phylogenomic reconstruction was performed using MEGA 11 [30] employing the Maximum Likelihood (ML) method under the General Time Reversible model with Gamma-distributed rate variation among sites and a proportion of invariable sites (GTR + G + I). Node confidence was evaluated with 1000 non-parametric bootstrap replicates. The resulting phylogenetic tree was edited and annotated using the Interactive Tree Of Life (iTOL v6; https://itol.embl.de; accessed on 10 July 2026; EMBL, Heidelberg, Germany) online tool [36].

## 3. Results

### 3.1. Sequencing, Assembly, and General Features of the A. brassicae Mitogenome

**Figure 1:**
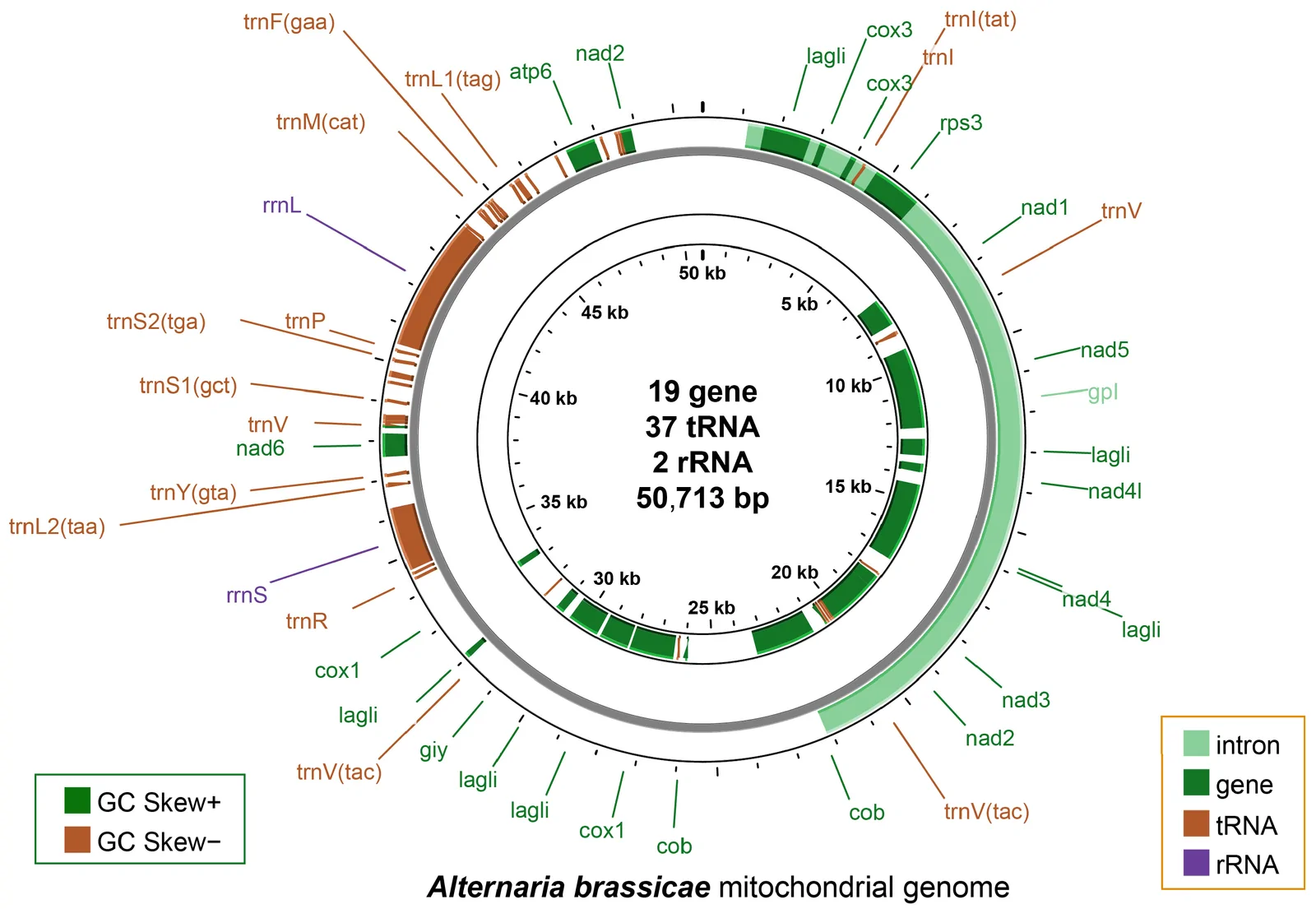
Circular map of the assembled and annotated *Alternaria brassicae* mitochondrial genome. Labels outside and inside the circle indicate features transcribed in opposite directions. Blue, orange, purple, and light green denote PCGs/iORFs, tRNAs, rRNAs, and predicted introns, respectively. The inner green and brown track shows positive and negative GC-skew. The scale is shown in kilobases; the center reports the assembly length and gene counts.

HiFi sequencing yielded a total of 1,385,133 clean reads, which were assembled into a circular mitochondrial DNA molecule of 50,713 bp in length (CNSA accession number: CNP0008733). The circularity of the mitochondrial contig was established by identifying identical terminal overlapping sequences generated by HiMT, which were then trimmed to linearize the molecule at the start of the rrnL (or cox1) gene. To independently validate assembly integrity and coverage, clean HiFi reads were mapped back to the finalized circular sequence using Minimap2 (v2.24). Per-base sequencing depth and mapping statistics were calculated using Samtools (v1.15), confirming a uniform average coverage of 309.0× across the entire mitogenome without structural breakpoints or coverage dropouts. Annotation results revealed that the mitochondrial genome contains 19 protein-coding genes: 12 named mitochondrial genes (*atp6*, *cox1*, *cox3*, *cob*, *nad1*, *nad2*, *nad3*, *nad4*, *nad4L*, *nad5*, *nad6*, and *rps3*) and 7 intronic ORFs (*lagli_0–lagli_5* and *giy*), together with 2 rRNA genes (*rrnL* and *rrnS*) and 37 tRNA genes (Table 1; Figure 1). Inspection of the predicted Cob sequence showed glycine at codon 143, and no Group I intron was inserted immediately after codon 143.

The overall GC content was 29.3%. A total of 33 SSR loci were identified, spanning 1157 bp, with an average density of one SSR per 1.52 kb. Mono-nucleotide repeats were predominant (57.6%), which aligns with the general characteristics of mitochondrial genomes. Among these mono-nucleotide repeats, A/T repeats accounted for 18 loci, comprising 94.7% of this type, strongly consistent with the typically high AT content of mitochondrial genomes. Furthermore, the core repeat length was primarily distributed within 10–12 bp (24 loci, 72.7%). The longest repeat unit was 140 bp (located at positions 45,171–45,310). Tandem repeat analysis indicated a high density of repeats in the 33–40 k and 45–50 k regions, which is fully consistent with the SSR analysis results. A notable long repeat unit of 132 bp, present in two copies, was identified at positions 23,604–23,873. Analysis using BiBiServ showed the presence of 44 direct repeats (12.22%), 165 inverted repeats (45.83%), 109 complementary repeats (30.28%), and 42 palindromic repeats (11.67%). Intron-containing structures were predicted in the *nad4*, *nad4L*, *nad5*, *cox3*, *cob*, and *cox1* genes; these predictions were not experimentally confirmed with transcript data (Figure 1).

### 3.2. Phylogenetic and Comparative Mitochondrial Genomic Analyses

**Figure 2:**
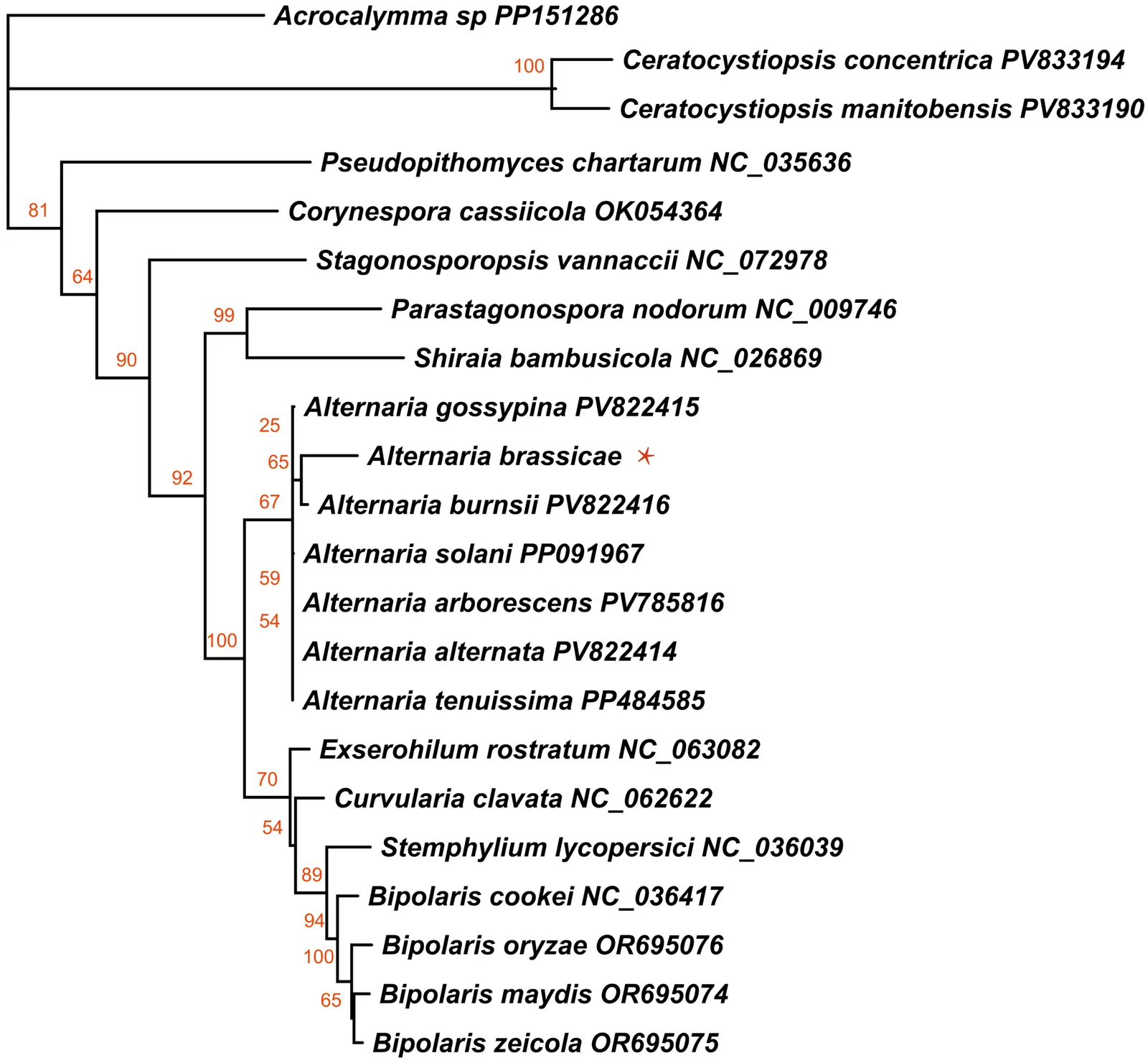
Maximum-likelihood tree inferred from concatenated codon-aligned nucleotide sequences of 12 mitochondrial PCGs from 22 fungal mitogenomes. The analysis used the GTR + G + I model and 1000 bootstrap replicates; bootstrap percentages are shown in orange at internal nodes. *Acrocalymma* sp. PP151286 was used to root the displayed topology. GenBank accession numbers follow the taxon names, and the red star marks *Alternaria brassicae*.

Phylogenetic analysis placed *Alternaria brassicae* (CNP0008733) as sister to *A. burnsii* (PV822416) with 65% bootstrap support; the grouping of this pair with *A. gossypina* had 25% support (Figure 2). These values indicate moderate support for the sister pair and weak support for the deeper grouping. Compared with the published *A. tenuissima* mitogenome (57,475 bp, 34 tRNAs, and two rRNAs), the *A. brassicae* assembly is 6762 bp shorter, contains three additional tRNAs, and has the same number of rRNAs [37]. A gene-order comparison was not performed in this study; therefore, no conclusion about conserved synteny is drawn.

## 4. Discussion

This study presents the first complete mitochondrial genome of *Alternaria brassicae* (50,713 bp, GC content 29.3%), encoding a conserved set of 19 protein-coding genes, 2 rRNAs, and 37 tRNAs. Compared to other previously published Alternaria mitogenomes, such as *A. alternata* (60.0 kb) and *A. solani* (58.1 kb), *A. brassicae* exhibits a relatively compact circular architecture [38]. Comparative evaluations indicate that this size variation across the genus is primarily driven by the dynamic expansion of non-coding intergenic spacers and intron populations rather than alterations in core coding regions [39]. Genome-wide repeat profiling identified 33 SSR loci and 360 interspersed repeats, representing candidate regions where repeat-length variation could arise through slipped-strand mispairing [40]. While actual polymorphism levels remain to be evaluated across geographic isolates, these repeat motifs provide candidate targets for developing molecular markers for population genetics. Additionally, whether these repeat-dense clusters mediate intramolecular recombination in *A. brassicae* warrants future experimental investigation [41].

Structural analysis predicted intron-containing structures in six respiratory genes (*nad4*, *nad4L*, *nad5*, *cox1*, *cox3*, and *cob*), consistent with intronic mobile elements reported in fungi [42]. These predictions were not validated with transcript data. The predicted Cob sequence retains G143, and no Group I intron occurs immediately after codon 143. These observations provide a sequence baseline for monitoring target-site mutations, but they do not demonstrate fungicide sensitivity without phenotypic testing [43]. Furthermore, phylogenomic reconstruction from codon-aligned nucleotide sequences of 12 mitochondrial PCGs placed *A. brassicae* as sister to *A. burnsii*, but the 65% bootstrap value indicates moderate rather than strong support. This mitochondrial result can be compared with nuclear-genome and multilocus classifications of *Alternaria* [44,45]. Overall, this complete mitogenome enriches organellar genomic resources for Dothideomycete plant pathogens, offering novel insights into fungal evolutionary biology, mitochondrial inheritance, and disease control strategies.

## 5. Conclusions

This study reports the first complete mitochondrial genome of *Alternaria brassicae*. The circular genome is 50,713 bp in length and encodes 19 protein-coding genes, 37 tRNAs, and 2 rRNAs. Phylogenetic analysis clarified the species’ taxonomic position within the genus and revealed characteristic structural features of its mitogenome. These results provide a foundational genomic resource for future studies on the population genetics, molecular diagnostics, and fungicide-resistance-associated variation in *A. brassicae*.

## Figures and Tables

**Table 1:** Annotation of the complete mitochondrial genome of *Alternaria brassicae*.

Functional Category	Gene Count	Gene Name(s)
Complex I (NADH dehydrogenase)	7	*nad1*, *nad2*, *nad3*, *nad4*, *nad4L*, *nad5*, *nad6*
Complex III (Ubiquinol-cytochrome c reductase)	1	*cob*
Complex IV (Cytochrome c oxidase)	2	*cox1*, *cox3*
Complex V (ATP synthase)	1	*atp6*
Ribosomal protein subunit	1	*rps3*
Subtotal (Core PCGs)	12	—
Intronic ORFs (iORFs)	7	*lagli_0*, *lagli_1*, *lagli_2*, *lagli_3*, *lagli_4*, *lagli_5*, *giy*
Total Protein-Coding Genes (PCGs)	19	—
Ribosomal RNAs (rRNAs)	2	*rrnL*, *rrnS*
Transfer RNAs (tRNAs)	37	20 amino acid types
Total Mitogenome Genes	58	—

Note: PCGs: Protein-coding genes; iORFs: Intronic open reading frames encoding putative LAGLIDADG or GIY-YIG family homing endonucleases; rRNA: Ribosomal RNA; tRNA: Transfer RNA.

## Data Availability

The assembled mitochondrial genome of *Alternaria brassicae* has been deposited in the China National GeneBank DataBase (CNSA; https://db.cngb.org/cnsa/; accessed on 10 July 2026) under the accession number [CNSA accession number: CNP0008733]. The raw PacBio HiFi reads analyzed in this study are publicly available from the NCBI Sequence Read Archive (https://www.ncbi.nlm.nih.gov/sra; accessed on 10 July 2026) under accession SRR32051149. All data generated in this study are available from the corresponding author upon reasonable request.

## Abbreviations

The following abbreviations are used in this manuscript:

rRNA	Ribosomal RNA
SRA	Sequence Read Archive
SSR	Simple sequence repeat
tRNA	Transfer RNA
MAFFT	Multiple alignment using fast Fourier transform
MEGA	Molecular evolutionary genetics analysis
MISA	Microsatellite identification tool
